# Retrospective cohort study of video-assisted thoracoscopic precise positioning reduction and internal fixation and thoracotomy reduction and internal fixation in treating multiple rib fractures

**DOI:** 10.12669/pjms.40.8.9196

**Published:** 2024-09

**Authors:** Jinlong Shi, Jing Liu

**Affiliations:** 1 Jinlong Shi Department of Cardio-Thoracic Surgery, The First People’s Hospital of Jingzhou, Jingzhou Hubei 434000, China; 2 Jing Liu Department of Cardio-Thoracic Surgery, The First Affiliated Hospital of Yangtze University, The Second Hospital of Jingzhou, Jingzhou Hubei 434000, China

**Keywords:** Internal fixation, Interleukin, Video-assisted thoracoscopic, Rib fracture, Postoperative recovery

## Abstract

**Objective:**

This study aimed to assess the clinical effect of video-assisted thoracoscopic precise positioning reduction and internal fixation and thoracotomy reduction and internal fixation in the therapy of multiple rib fractures.

**Methods:**

A total of 80 patients with multiple rib fractures in First People’s Hospital of Jingzhou from January 2021 to December 2022 were separated into control group (CG, 40 cases) and research group (RG, 40 cases) by random number table method. Patients in the CG received thoracotomy reduction and internal fixation. Patients in the RG received video-assisted thoracoscopic precise positioning reduction and internal fixation. Surgery-related indexes, serum interleukin, visual analogue scale (VAS) was used to evaluate postoperative pain, arterial oxygen saturation (SaO2) and oxygenation index (PaO2/FiO2), and pulmonary function indexes were measured in the two groups.

**Results:**

After surgery, Visual analog scale (VAS) scores at 24 hour after surgery were reduced compared to six hour and 12 hour after surgery (P<0.05). VAS score at 12 hour after surgery was increased relative to 6 h after surgery (P<0.05), and VAS score at six hour, 12 hour as well as 24 hour after surgery in the RG was lessened in comparison with the CG (P<0.05). One day after surgery, SaO_2_ and PaO_2_/FiO_2_ in two groups were elevated compared to before surgery, and those in RG was increased in contrast to the CG (P<0.05). forced expiratory volume (FEV1), forced vital capacity (FVC), peak expiratory flow velocity (PEF) along with FEV1/FVC levels in two groups were increased (P<0.05).

**Conclusion:**

Compared with thoracotomy reduction and internal fixation, video-assisted thoracoscopic accurate positioning and internal fixation in treating patients with multiple rib fractures had a short treatment time, obvious advantages, and strong feasibility.

## INTRODUCTION

Multiple rib fracture is a serious traumatic fracture of the chest, with multiple fracture sites and broken ends that can poke multiple organs or cause chest wall softening, seriously affecting patients’ circulatory respiratory function, and often combined with pneumothorax, lung contusion and laceration and other injuries.^1^,^2^ If the treatment is not active and rapid, these complications affect the recovery of lung function and prolong the hospital stay, and even lead to respiratory and circulatory disorders and life-threatening. Therefore, timely and appropriate treatment plays a very important role in patients with multiple rib fractures.^3^ In the treatment of multiple rib fracture, conservative treatment or reduction and internal fixation are mainly given according to the specific conditions of the patient.^4^ Due to the poor fixation effect of conservative treatment on the proximal end of fracture and the long recovery time, the clinical application is limited. The technique of reduction and internal fixation has high stability performance and is more beneficial to the recovery of normal structure and function of the chest, with obvious effects.^5^ In the past, thoracic reduction and internal fixation were mainly applied in treating multiple rib fracture, but the trauma to patients was large and the postoperative recovery time was long.^6^

Recently, with the continuous development of minimally invasive technology, video-assisted thoracoscopy has been extensively used in various thoracic surgeries. It can be used to explore the injured part and repair tissues and organs. Studies have shown that is uniportal thoracoscopic pulmonary lobectomy has significant clinical efficacy in the treatment of lung cancer.^7^ Therefore, it is favored by doctors and patients due to its features of small incision, small damage, and quick recovery.^8^, ^9^ However, there are few reports on the use of video-assisted thoracoscopy in the reduction and internal fixation of multiple rib fractures. Therefore, the aim of this research was to probe the clinical effect of video-assisted thoracoscopic precise positioning reduction and internal fixation in the treatment of multiple rib fractures, so as to provide a clinical basis for the treatment of multiple rib fractures and reduce the pain and length of hospital stay for patients.

## METHODS

A total of 80 patients with multiple rib fractures admitted to our hospital from January 2021 to December 2022 were chosen as the study objects. All subjects were divided into control group (CG, 40 cases) as well as research group (RG, 40 cases) by random number table method. In the CG, 23 males together with 17 females were contained. The mean age was 46.28±4.36 years. Rib fracture 3-6. The causes of injury included 15 traffic injuries, 12 fall injuries, eight heavy object injuries, and five other injuries. In the RG, 25 males together with 15 females were included, with an average age of 46.42±4.37 years. Rib fracture 3-7. The causes of injury included traffic injuries in 16 cases, falling injuries from heights in 13 cases, heavy objects in seven cases, and other four cases. No significant difference was discovered in the general data between two groups (P>0.05), indicating comparability.

### Ethical Approval:

This study was approved by the medical ethics Committee of First People’s Hospital of Jingzhou, Ethics number JZLL002 (A), and all patients had signed informed consents.

### Inclusion criteria:

- Patients and their family members were aware of the study and voluntarily participated in it.
- Complete information.
- Stable vital signs, stable venous access or maintain effective circulation.
- The time of injury was less than 12 hours.

### Exclusion criteria:

- Combined with severe liver and kidney failure.
- Suffered from chronic lung disease or severe lung infection.
- Patients with lung cancer or other malignant tumors, whose survival time was less than six months.
- Family members did not support participation.

### Methods:

All subjects underwent routine CT examination, dilatation, oxygen inhalation and infection prevention after admission. Patients in the CG were treated with thoracotomy reduction and internal fixation^10^: three-dimensional CT images were established according to the clinical symptoms and examination results of the patients, the range and degree of fracture were determined, the internal fixation operation plan was developed, and the titanium nickel alloy bone plate used in the operation was immersed in ice physiological saline.

The incision site was set and marked before surgery, endotracheal intubation anesthesia was performed, the patient was put in lateral decubitus position, combined with the fracture situation, a 5-7 cm incision was made to completely expose the fracture part, and after routine cleaning of blood collection and clots at the broken bone site, the periosteum at the rib fracture (about 3 cm in length) was peeled, the bone fracture site was reset (pay attention not to damage the pleura), and the cooled titanium nickel alloy bone plate was molded according to the shape of the broken bone, in the shape of an arm-shaped ring, and fix it to the broken end of the rib. Then gauze soaked with 40-45 °C normal saline was used to apply hot compress to the bone plate until fixed firmly. Postoperative thoracic drainage tube was placed and the incision was sutured.

Patients in the RG were treated with video-assisted thoracoscopic precise positioning reduction and internal fixation: preoperative operation plan was made and bone plate was prepared, etc. Patients were placed in lateral decubation and endotracheal intubation was performed under general anesthesia. After the effect of anesthesia, a 15 mm long incision was made between the 7th ribs of the midaxillary line on the affected side, and thoracoscopy was placed to explore the specific situation in the pleural cavity, clean the blood collection as well as clots at the bone fracture site, and electrocoagulation was performed to stop the blood. The periosteum of the broken end of the rib was dissected using video-assisted thoracoscopy, and the periosteum of the broken end of the rib was dissected about 3 cm. The fracture reduction and fixation methods were the same as those of the CG. Both groups were given fluid rehydration, antibacterial drugs to prevent infection and analgesic drugs.

### Observation indicators:

- ***Surgery-related indicators:*** The operation time, intraoperative blood loss, respiratory assistance time, chest tube drainage volume, time to get out of bed, and the number of analgesic uses were compared between two groups.
- Fasting peripheral venous blood was collected before and one day after surgery, and the upper serum was separated by centrifugation. Serum interleukin-6 (IL)-6, IL-8, as well as IL-1β in two groups were detected by enzyme-linked immunosorbent assay. According to the instructions of the kit, two groups of serum IL-6, IL-8 and IL-1β were detected by enzyme-linked immunosorbent assay. The relative ELISA kits were all purchased from Elabscience (E-EL-H6156, E-EL-H6008, E-EL-H0149)
- ***Postoperative pain degree changes:*** Visual analog scale (VAS)^11^ was implemented to assess the postoperative pain at six hours, 12 hours, along with 24 hours.
- Blood gas samples were collected from the radial or femoral arteries before surgery and one day after surgery. Arterial oxygen saturation (SaO2) together with ox
- ***Changes in lung function indexes:*** AS-507 pulmonary function detector provided by Shanghai Hanrong Medical Instrument Co., LTD. was used before and after treatment to detect 1-second forced expiratory volume (FEV1), forced vital capacity (FVC) along with maximum peak expiratory flow velocity (PEF), and to calculate FEV1/FVC.
- ***Incidence rate of complication:*** The number of complications (pulmonary infection, pneumothorax, atelectasis, and h
- ***Post-operation follow-up:*** All patients were followed up for 3-6 months and underwent imaging examination.

### Statistical analysis:

SPSS 19.0 statistical software was implemented for analysis. t test was adopted for measurement data and χ^2^ test was adopted for counting data. P<0.05 was statistically significant.

## RESULTS

### Comparison of Surgery-related indexes between GC and RC:

**Fig.1:**
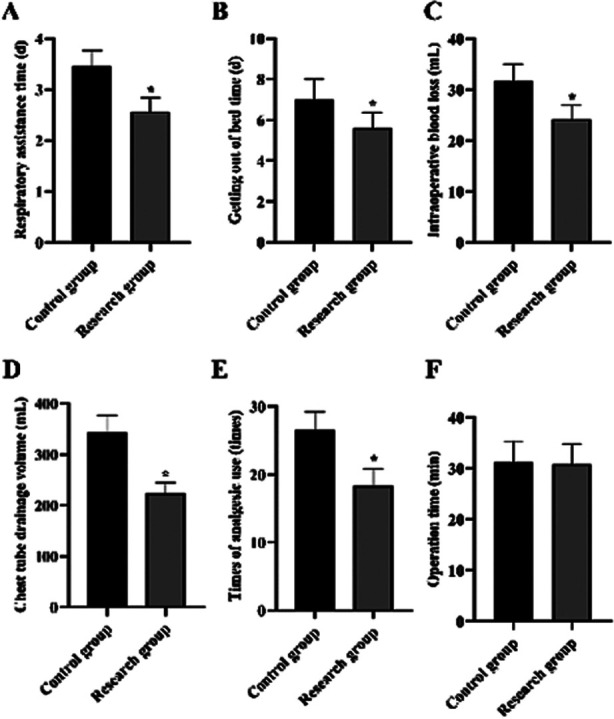
Surgery-related indexes in 2 groups. (A) The respiratory assistance time of the RG and CG. (B) The getting out of bedtime of the RG and CG. (C) The intraoperative blood loss of the RG and CG. (D) The chest tube drainage volume of the RG and CG. (E) The times of analgesic use of the RG and CG. (F) The operation time of the RG and CG. *P<0.05.

It is displayed in Fig.1 that, the respiratory assistance time and getting out of bedtime in the RG were shorter than those in the CG (Fig.1(A-B)), and the intraoperative blood loss, chest tube drainage volume and the times of analgesic use in the RG were all shorter than those in the CG (P<0.05) (Fig.1(C-E)). No difference was discovered in operation time between two groups (P>0.05) (Fig.1F).

### Comparison of Interleukin levels between GC and RC:

**Fig.2:**
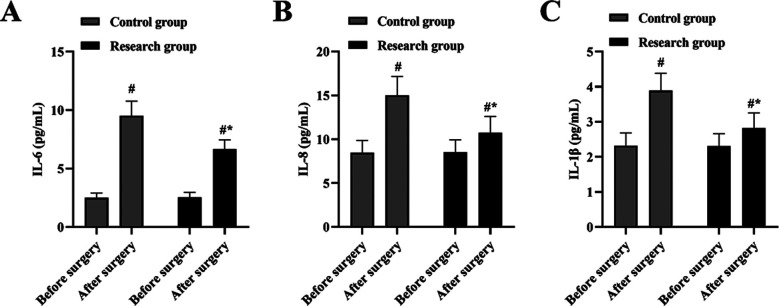
Interleukin levels in 2 groups. (A) The IL-6 level of CG and RC after and before surgery. (B) The IL-8 level of CG and RC after and before surgery. (C) The IL-1β level of CG and RC after and before surgery. #P<0.05, compared with before surgery, *P<0.05, compared with the control group.

It is highlighted in Fig.2 that, no difference was discovered in the level of interleukin (IL-6, IL-8 and IL-1β) between CG and RG groups before surgery (P>0.05) (Fig.2(A-C)). In both the GC and the RC, the IL-6, IL-8 and IL-1β level after surgery was higher than before surgery (P<0.05) (Fig.2(A-C)).IL-6, IL-8 as well as IL-1β levels in RG was decreased, compared to the CG one day after surgery (P<0.05) (Fig.(2A-C)).

### Comparison of Postoperative pain degree changes between GC and RC:

**Fig.3:**
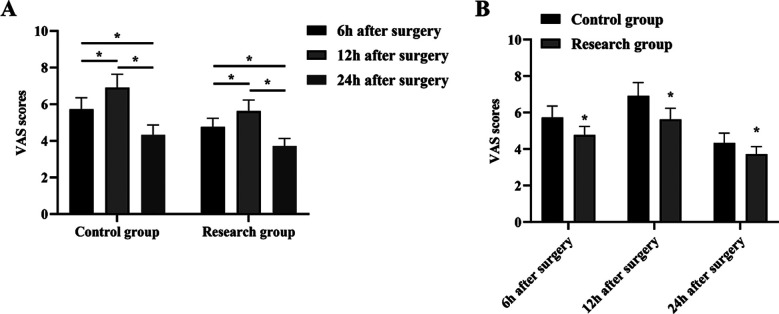
Postoperative pain degree changes in 2 groups. (A) VAS scores were compared at 6 hours, 12 hours, and 24 hours after surgery in GC and RC. (B) The VAS scores of GC and RC were compared at 6 hours, 12 hours, and 24 hours after surgery, respectively. *P<0.05.

It is highlighted in Fig.3 that, VAS scores at 24 hours after surgery were reduced compared to six hours as well as 12 hours after surgery (P<0.05) (Figure 3A). The VAS score at 12 hours after surgery was elevated compared to six hours after surgery (P<0.05) (Figure 3A), and the VAS score at 6 hours, 12 hours as well as 24 hours after surgery in the RG was decreased relative to the CG (P<0.05) (Figure 3B).

### Comparison of Blood gas index between GC and RC:

**Fig.4:**
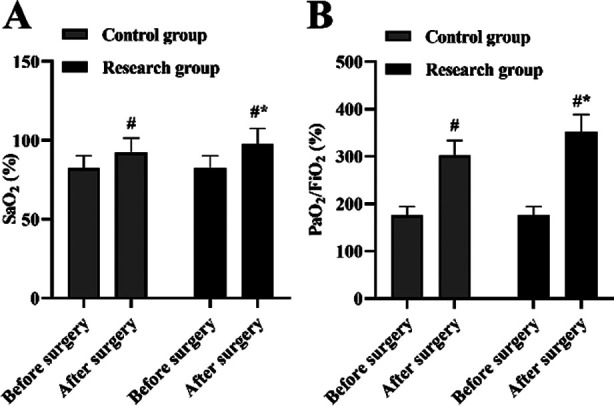
Blood gas index in two groups. (A) The SaO_2_ level of CG and RC after and before surgery. (B) The PaO_2_/FiO_2_ level of CG and RC after and before surgery. #P<0.05, compared with before surgery, *P<0.05, compared with the control group.

It was demonstrated in Fig.4 that, one day after surgery, SaO_2_ and PaO_2_/FiO_2_ in two groups were elevated compared to before surgery, and those in the RG was increased relative to the CG (P<0.05) (Figure 4A-B).

### Changes of lung function index between GC and RC:

**Fig.5:**
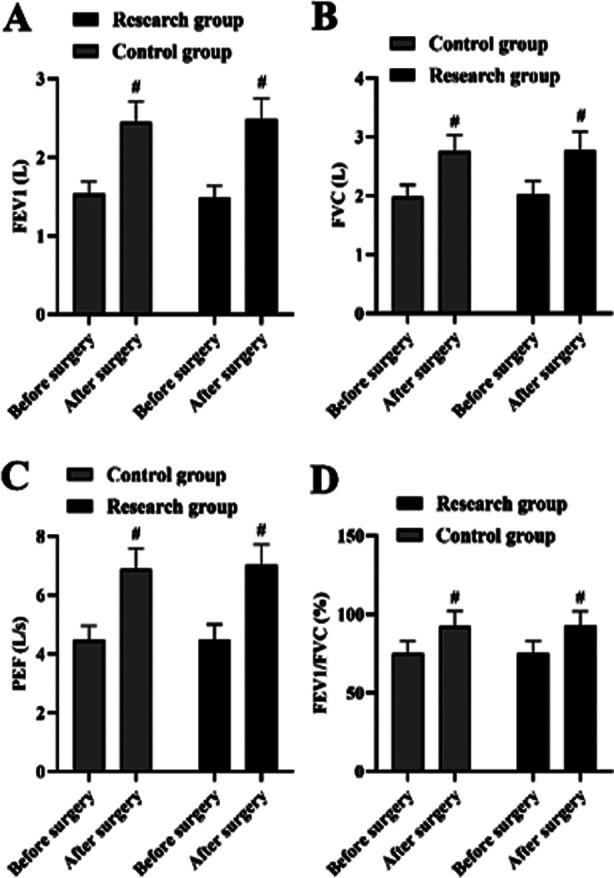
Changes of lung function index in 2 groups. (A) The FEV1 level of CG and RC after and before surgery. (B) The FVC level of CG and RC after and before surgery. (C) The PEF level of CG and RC after and before surgery. (D) The FEV1/FVC level of CG and RC after and before surgery. #P<0.05, compared with before surgery.

It was revealed in Fig.5 that, prior to surgery, no statistical significance was discovered in FEV1, FVC, PEF along with FEV1/FVC levels between two groups (P>0.05) FEV1, FVC, PEF together with FEV1/FVC levels in two groups were increased after surgery relative to those before surgery (P<0.05). No significant differences were discovered in FEV1, FVC, PEF along with FEV1/FVC levels between two groups after surgery (P>0.05) (Figure 5A-D).

### Incidence of complication in GC and RC:

In the GC and RC groups, we assessed associated postoperative complications including pulmonary infection, pneumothorax, atelectasis, hemothorax, and total incidence rate. The results showed that the total incidence rate complication of the RG was lessened compared to the CG (P<0.05, Table-I).

**Table-I:** Incidence of complication in two groups.

Groups	Pulmonary infection	Pneumothorax	Atelectasis	Hemothorax	Total incidence rate
Control group (n=40)	2	3	2	2	9 (22.50%)
Research group (n=40)	1	0	1	0	2 (5.00%)
*χ* ^2^	5.17				
*P*	<0.05				

## DISCUSSION

The key to multiple rib fracture treatment is to improve and stabilize the chest wall, relieve chest pain, restore respiratory function, and take effective fixation.^12^ With the usual conservative treatment, although the operation is simple, but the recovery time is long, and the external fixation stability is poor.^13^ When patients breathe, the thoracic movement will also lead to increased pain, affect the normal cough, expectoration, as well as increase the occurrence of lung infection as well as other complications.^14^ Thoracotomy and video-assisted thoracotomy are commonly used in treating patients with rib fractures.^15^ Thoracotomy is simple in operation, and the removal of blood and clots is complete, but the incision is large, the trauma is great, the inflammatory response is significant and the recovery time is long.^16^,^17^ Video-assisted thoracotomy surgery is a minimally invasive operation, but it is complicated and requires high technical requirements for the operator.^18^

The outcomes of this study showed that the breathing assistance time and getting out of bedtime of patients in the RG were shorter compared to the CG, and the intraoperative blood loss, chest tube drainage volume and times of analgesic use in the RG were less than those in the CG, and there was no statistical significance in the operation time between two groups. VAS score at 6 hours, 12 hours, and 24 hours after surgery in the RG was lower than that in the CG. SaO_2_ and PaO_2_/FiO_2_ on the one day after surgery in two groups were higher than those before surgery, and those in RG was higher than the CG.

These results indicated that the use of video-assisted thoracoscopic accurate positioning reduction and internal fixation in the treatment of patients with multiple rib fractures was helpful to shorten the recovery time of patients. It also reduce the surgical damage to the body, and thus reduce the postoperative pain and complication rate, and improve the safety of treatment. Traditional thoracotomy has large incision, more intraoperative blood loss, longer recovery time, and more intraoperative exposed sites, which leads to higher postoperative pain and complication rate. United States Veterans report showed that Video-assisted thoracoscopic surgery can greatly reduce the postoperative pain and complication rate of patients, and promote the rehabilitation process of patients, with significant advantages.^19^ Previous clinical studies have reported that 42 patients with multiple rib fractures underwent single-operative port full VATS treatment, which was clinically effective and feasible.^9^ This study further expanded the sample range and proved that video-assisted thoracoscopy has significant significance for the clinical treatment of multiple rib fractures.

Cytokines play an induction role in immune response and inflammatory response, and IL-6, IL-8 and IL-1β are important interleukins.^20^ The outcomes of this research showed that the level of interleukin was elevated in two groups after surgery, but those in the RG were declined compared to the CG. The results also showed that video-assisted thoracoscopic precise positioning surgery caused less trauma to the body and could reduce the inflammatory response of the body, so as to accelerate postoperative recovery and reduce postoperative complications.

After multiple rib fractures, the chest wall softens and collapses due to loss of support, and the integrity of the thoracic tissue is damaged, and then abnormal respiratory movement occurs.^21^ Patients also stimulate the intercostal nerve to produce respiratory pain due to progressive displacement factors such as abnormal respiratory movement when the mediastinum swings back and forth with breathing, and the fracture end rubs back and forth, which affects the tidal volume, and the lung function damage is more serious for patients with pulmonary contusion.^22^

In this research, the levels of lung function indexes FEV1, FVC, PEF as well as FEV1/FVC were detected before and after surgery, and the outcomes indicated that prior to surgery, no statistical significance was discovered in FEV1, FVC, PEF along with FEV1/FVC levels between two groups. FEV1, FVC, PEF together with FEV1/FVC levels in two groups were increased after surgery relative to those before surgery. No significant differences were discovered in FEV1, FVC, PEF along with FEV1/FVC levels between two groups after surgery.

Early fracture fixation surgery can improve pain and ventilation and prevent complications from chest trauma. Our study has suggested that both thoracotomy reduction and internal fixation and video-assisted thoracoscopic precise positioning surgery could effectively restore patients’ respiratory function in patients with multiple rib fractures and slow the inflammatory response. Additionally, all patients were followed up for 3-6 months after surgery, and imaging examinations showed good rib alignment, callus formation and healing, and no loosening or fracture of rib plate, which suggested that both thoracotomy reduction and internal fixation and video-assisted thoracoscopic precise positioning surgery had obvious therapeutic effect on treating patients with multiple rib fractures

### Limitations:

This study still needs to expand the sample size to reduce the bias of the results, and in future studies we will follow patients for more long-term clinical outcomes.

## CONCLUSION

Compared with thoracotomy reduction and internal fixation, video-assisted thoracoscopic accurate positioning and internal fixation in treating patients with multiple rib fractures had a short treatment time, light damage to patients, and no impact on lung function of patients, showing reasonable safety, obvious advantages, and strong feasibility.

### Recommendation:

Video-assisted thoracoscopic precision positioning surgery is less invasive and has a rapid postoperative recovery, so it is worthy of clinical use. In addition, fractures in areas that are difficult to fix, such as armpits, shoulder blades, and female breasts, can also be explored in the future.

### Author’s Contribution:

**JL** and **JS:** Made equal contributions in study design, data collection, statistical analysis, figure preparation, manuscript draft and revision. **JL** and **JS:** Are responsible and accountable for the accuracy or integrity of the work.
